# Community Health Status Indicators Project: The Development of a National Approach to Community Health

**Published:** 2008-06-15

**Authors:** Marilyn Metzler, Norma Kanarek, Keisher Highsmith, Roger Straw, Ron Bialek, Jennifer Stanley, Ione Auston, Richard Klein

**Affiliations:** Centers for Disease Control and Prevention/McKing Consulting; Johns Hopkins Bloomberg School of Public Health (formerly of the Public Health Foundation), Baltimore, Maryland; Health Resources and Services Administration, Rockville, Maryland; Health Resources and Services Administration, Rockville, Maryland; Public Health Foundation, Washington, District of Columbia; Public Health Foundation, Washington, District of Columbia; National Library of Medicine, Bethesda, Maryland; Centers for Disease Control and Prevention, Hyattsville, Maryland

## Abstract

The Community Health Status Indicators Project (CHSI) 2008 provides 16-page reports for the 3141 counties in the United States, each of which includes more than 300 county-specific data items related to chronic and infectious diseases, birth characteristics or outcomes, causes of death, environmental health, availability of health services, behavioral risk factors, health-related quality of life, vulnerable populations, summary measures of health, and health disparities. The CHSI, originally initiated in 2000, provides county-level health profiles for all U.S. counties so that programs addressing community health can readily access community health indicators. Each county report also permits comparisons of a county's health status with similar "peer counties," with all counties, and with national *Healthy People 2010* objectives. Under the leadership of a public–private partnership, the CHSI Steering Committee updated each county report and added new information and features to create CHSI 2008. This new CHSI version includes data for 1994 through 2006 from multiple surveillance systems. New features include an enhanced Web site, an Internet mapping application, and a downloadable database of the indicators for all counties.

## Introduction

The *Healthy People 2010* goals to eliminate health disparities and improve length and quality of life (1) have become the central focus of many public health activities, increasing the emphasis on community-based approaches to health improvement. This shift to broader and more local approaches requires the development of new strategies, tools, and resources that are responsive to the needs of communities. Foremost among these is the need for relevant data that communities can use to assess and monitor local health and to guide program and policy development. Rapid advances in technology allow for increased access to data, but data are often difficult to locate, and methodology, technology, and proprietary barriers between users and providers often make it difficult to link or combine disparate data sets for use at the local level (2). Many communities have made efforts to respond to local data needs (3). In addition, several cross-community initiatives, including the National Neighborhood Indicators Partnership (4), the Missouri Information for Community Assessment Priority Setting Model (5), and the CDC-sponsored Community Assessment Initiative (6), have made significant contributions to the knowledge base on community indicators and local health. However, a comprehensive, systematic initiative for communities unable to secure local data is also needed. Such an initiative could serve as a national resource for conducting comparisons across communities and as a public health performance-monitoring system, and stimulate the development of measures that enhance the national information network and processes that improve local conditions for health (7).

## Background

The Health Resources and Services Administration (HRSA) initiated a pilot of the Community Health Status Indicators Project (CHSI) in 1998 to provide health indicator data at the county level. By 2000, HRSA, working with the Public Health Foundation (PHF), the Association of State and Territorial Health Officials (ASTHO), and the National Association of County and City Health Officials (NACCHO), developed reports on the health status of each county in the United States (8). The CHSI reports provide a profile of each county's overall health status using a broad spectrum of indicators. The reports allow each county to compare its indicators to *Healthy People 2010* targets, to indicators for the United States overall, and to  indicators for peer counties (counties that share selected demographic characteristics). The CHSI 2000 reports were developed primarily for public health professionals and community planners to use as a tool for setting priorities and targeting resources to improve community health. In August 2000, the CHSI Steering Committee distributed profiles for 3082 U.S. counties, with data from 1988 through 1998, to every state and local health agency in the United States. The CHSI developers also made the profiles available on the Internet. In 2002, the Web-based resource was discontinued, but the PHF made the CHSI 2000 county reports and other project products available for purchase on CD-ROM.

Recognizing the value of the CHSI in an era of rapidly expanding community health initiatives, a group of federal and private partners convened as a steering committee in 2004 to evaluate, update, and further develop the CHSI. The result of this effort is referred to here as CHSI 2008. Federal partners on the CHSI 2008 Steering Committee were HRSA, the Centers for Disease Control and Prevention (CDC), and the National Library of Medicine (NLM). Private partners include representatives from the PHF and faculty from Johns Hopkins University, and advisory partners were NACCHO, ASTHO, and the National Association of Local Boards of Health (NALBOH). The CHSI 2008 Steering Committee has also developed relationships with other indicator initiatives, including the Community Indicators Consortium (9) and the National Infrastructure for Community Statistics of the Brookings Institution (10).

## CHSI 2008: Updated and Enhanced

In response to recommendations from a user evaluation of CHSI 2000, the CHSI 2008 steering committee retained the original indicators and report format. This enabled the committee to use available resources to update existing data elements and to develop new features to improve access to and usability of the Web-based format. New partnerships have been established to support regular updates, increased utility, and long-term sustainability of the project, which CHSI 2000 users also noted as important. Following is a description of the updated and enhanced CHSI 2008.

### Community profiles

CHSI 2008 profiles provide a county-specific report for every U.S. county and a small number of independent cities. A 16-page report with more than 300 county-specific data items provides a comprehensive and comparable snapshot of each county's health. The steering committee originally selected indicators from CDC's consensus indicators of health (11), *Healthy People 2000 *(12), early drafts of *Healthy People 2010* (13,14), and expert opinion from the CHSI 2000 Advisory Group. Indicators were chosen on the basis of the following characteristics: the indicators were important to public health, were actionable, were regularly reported, and were available for all U.S. counties. The indicators address chronic and infectious diseases, birth characteristics or outcomes, causes of death, environmental health, availability of health services, behavioral risk factors, vulnerable populations, summary measures of health (health-related quality of life, life expectancy, and all-cause mortality), health disparities, and the relative importance of a subset of indicators. Data are from CDC's National Vital Statistics System, Behavioral Risk Factor Surveillance System (BRFSS), and Infectious Diseases Reporting System; the Environmental Protection Agency's Air Quality Reporting System and Toxic Release Inventory; the U.S. Bureau of the Census's Bureau of Labor Statistics and Current Population Survey; HRSA; the U.S. Substance Abuse and Mental Health Services Administration; the Centers for Medicare and Medicaid Services; and the Harvard Initiative for Global Health (15).

CHSI 2008 profiles include data from 1994 through 2006 for 3141 counties. The most recently available data are aggregated over the last 3-, 5-, or 10-year period, depending on county population, to optimize the reliability of the estimates. CHSI 2000 reported most of the BRFSS information and health insurance coverage estimates for states rather than counties; CHSI 2008 provides county-specific estimates for most local jurisdictions. Where applicable,* Healthy People 2010* definitions were used for the indicators. Mortality rates are age-adjusted to the 2000 standard population (16). Rules for suppressing estimates based on small numbers were implemented. Confidence intervals for BRFSS and vital statistics data are provided on the Web site; confidence intervals for vital statistics data are also included in the reports.

Each county report compares the local county's health status with that of national *Healthy People 2010* objectives and that of peer counties for the same time period. Peer counties are defined by several demographic characteristics and may be inside or outside the same state. There are 88 peer county groupings, with an average of 35 counties in each group (range = 14–58). Peer groupings were determined by a hierarchical segmentation of counties. First, counties were grouped according to frontier status (fewer than 7 people per square mile or more than 7 people per square mile) and population size (7 categories, ranging from <25,000 to >1,000,000). As the number of counties in each category allowed, further groupings were defined by poverty (quartiles ranging from ≤10.55% to >19.26%), age (percentage of county residents aged <18 years or ≥65 years), and population density (measured in half deciles). In CHSI 2000, only state-level data were available for Alaska, which does not have counties. At the time the CHSI 2000 reports were generated, data for individual Virginia cities were also lacking; therefore, these cities were grouped with their surrounding or adjacent counties. Since 2000, Alaska has defined aggregates that function much like counties. On the basis of feedback from the CHSI evaluation, the Alaska aggregates and Virginia independent cities' data have been assigned to appropriate peer groupings in CHSI 2008. For additional information on the development of the CHSI peer groupings, see Kanarek et al in this issue of *Preventing Chronic Disease* (17).

The CHSI also presents the vital statistics indicators, using a 2-by-2 comparative health importance table illustrating county health status compared with peers and with the United States overall. Highest priority county health conditions are shown, relative to the medians, as worse than their peers and worse than the national rate. Lowest priority conditions are those with indicators better than their peers and better than the national rate. An apple is used to indicate county standing more favorable or equal to peers or to U.S. median values — an indication of "health." A magnifying glass indicates county standing less favorable than peers or U.S. median values — an indication to "take a closer look." The auxiliary document, "Data Sources, Definitions, and Notes: Community Health Status Indicators 2008 Report," makes available additional information on CHSI 2008 data sources, definitions of indicators, and methods used to calculate estimates (18).

### Enhanced Web site design

The CHSI Web site, http://communityhealth.hhs.gov, provides access to the formatted, printable CHSI 2008 reports for each county, with data from 1994 through 2006. Users can create maps displaying health indicator information. In addition, the indicators used in CHSI 2008 are available in a downloadable format with associated documentation of the data sources. The Web site allows users to visually compare any county with its peer counties and with U.S. *Healthy People 2010* targets. It provides charts and graphs illustrating county-specific rates of premature death and preventive services use. Public health officials, policy makers, community organizations, and the general public can use the information as a foundation for planning and action or for developing partnerships to address community health. Over time, additional resources, training and other support materials, links to related sites, and other information about health indicators will be added to the Web site.

### Geographic Information Systems (GIS) feature

To increase the range of options for analyzing, displaying, and understanding community health data, the CHSI 2008 Steering Committee convened a subcommittee to explore the development of an Internet mapping application. In addition to the CHSI 2008 Steering Committee and advisory partners, the subcommittee included CDC's Agency for Toxic Substances and Disease Registry and the Polis Center at Indiana University Purdue University, Indianapolis. The result of the subcommittee's work, the Community Health Status Indicators Geographic Information Systems Analyst (CHSI GIS Analyst) is an easy-to-use, Web-based mapping application that enables the user to visualize, explore, and understand the geography of county indicators. An indicator for any county can be mapped and compared visually with the same indicator for other areas, including peer counties and neighboring counties. CHSI GIS Analyst is accessible from the CHSI 2008 Web site (http://communityhealth.hhs.gov). For additional information on the development of CHSI GIS Analyst, see Heitgerd et al in this issue of *Preventing Chronic Disease* (19). Future plans call for creating an additional Web site dedicated to CHSI GIS Analyst, expanding its mapping capabilities, and developing tools and other resources to improve accessibility.

### Dissemination and evaluation

In addition to making CHSI 2008 available on a Web site, the steering committee and partners developed a multicomponent dissemination and evaluation plan, supported in part by the Robert Wood Johnson Foundation (RWJF). The plan includes announcements distributed through electronic and print newsletters, fact sheets, conference calls with member associations, announcements on partner organizations' Web sites, manuscripts, and presentations at national meetings.

Evaluation of CHSI 2008 will consist of multiple approaches, including tracking use of the reports and database and assessing user comments. ASTHO, NACCHO, and NALBOH will also survey their members to evaluate adoption and utility of CHSI 2008. Representatives from community-based organizations working to address health disparities at the local level will also provide feedback to increase the utility of the CHSI from their perspectives. Evaluation findings will be posted on the CHSI Web site. Additional dissemination and evaluation activities will be developed as interest expands and resources allow.

## Looking Toward the Future

The CHSI is a resource that was developed in the context of 1998 public health system and technology. In the intervening years, many factors have changed, including rapid advances in technology and expansion of the boundaries of the traditional public health system. The updated CHSI takes several of these changes into consideration. However, to provide a relevant, regularly updated community assessment resource, a number of challenges must be addressed.

### Expanding the range of partners

Expanding the range of partners is vital for the growth and development of the CHSI if it is to remain a relevant resource for assessing community health. Because government has a responsibility to promote and protect health, the founding CHSI partnership and products have focused on the perspectives and needs of local public health agencies. In order to update and enhance CHSI 2000 as quickly as possible with limited resources, the steering committee decided to involve in the current partnership a small but expanded set of federal and private partners whose primary mission is public health protection and promotion. For example, the addition of NLM to the CHSI partnership expanded the ability of the group to reach a large segment of the public health workforce, given NLM's role in training state and local public health officials in the use of available information resources applicable to public health (20-22). As an added benefit, NLM also partners with community librarians and community health workers to support the development and use of community health planning resources (23,24). The advisory partners for CHSI 2008 are the United Way, the Association for Community Health Improvement, representatives from local health assessment projects, representatives of the Racial and Ethnic Approaches to Community Health initiative, and members of NACCHO, ASTHO, and NALBOH.

The original CHSI 2000 Web site received 20,000 hits per month. This usage highlighted the value of providing community profiles to a wide range of users. Communities are settings in which social, economic, and physical environments affect health. Communities also are critical partners in the public health system through the participation and perspectives of community members, organizations, and networks. Bringing community perspectives to the partnership will provide important insight to guide the future development of the CHSI, including how to increase the usefulness of this resource in bringing about changes in local policies and systems.

Assuring health is a primary public health activity, but it cannot be accomplished by public health agencies alone. Government agencies and organizations with primary missions related to health (e.g., housing, employment) can facilitate access to relevant data and stimulate the development of new measures and collaborative activities. In addition, the CHSI steering committee is interested in promoting the use of the CHSI for community health research and for policy development that responds to local, regional, and national public health needs. Expanding the partnership to include researchers will facilitate building, consolidating, and sharing knowledge and expertise that contribute to health promotion and that strengthen community and public health research capacities. We hope that these capacities, together with scientific, technological, organizational, and educational innovations, will bring new energies and resources to meeting the goal of community health improvement.

### Understanding community context and health

*Healthy People 2010* acknowledges that improving social, economic, and physical environments is essential to increasing years of quality life and eliminating health disparities (1) and calls for the development of relevant indicators. However, these aspects of *Healthy People 2010* have received minimal attention (25). Recognition of the importance of addressing the social determinants of health (26) is increasing across the public health arena, as evidenced by the inclusion of social determinants within a key objective in CDC's Healthy People in Healthy Places goals (27), by CDC's convening of an expert panel on social determinants of health in April 2008, and by the World Health Organization's convening of its Commission on Social Determinants of Health (28). Although these activities and others demonstrate recognition of the importance of addressing conditions that have an impact on health, understanding of how community context influences health remains limited, in part because there is a dearth of relevant and timely measures for assessing this influence. The first official release, in 2008, of American Community Survey multiyear estimates for all U.S. counties will provide timely census data that previously were available only through the decennial census (29). These data will be used in future CHSI updates. A social context module under development that CDC will pilot test in its BRFSS will also provide important information for understanding individual health behaviors and the social conditions in which they occur.

By many measures, current public health surveillance systems are also limited in scope and precision by the absence of data on positive attributes of health as opposed to disease and by the lack of data on social factors that affect health (30). Increased availability of social and health indicators can support monitoring of indicators that make a difference to community health outcomes and help communities choose appropriate interventions and establish priorities for local programs and policies. In addition, having social and health indicators will allow investigators to continue development of conceptual framing to identify relationships between context and health (31); link epidemiologic and contextual data in innovative systems modeling (32); and stimulate research on the relationships between social determinants and health outcomes.

### Supporting and benefiting from methodological and technological advances

Access to timely, relevant data is critical for the future development of the CHSI. New technology will help improve timely access to data and improve ways in which data are gathered and managed. For example, the National Infrastructure for Community Statistics, under the sponsorship of the Brookings Institution, is supporting the development of a network to link and integrate national, regional, state, and local data across criminal justice, health, environment, environmental health, and geographic information systems (10). This network will reduce or eliminate the need for labor-intensive data warehouse development.

Equally important are methodological advances that increase the accuracy of local estimates. For example, Bayesian smoothing, a technique that involves borrowing data from surrounding areas, is one method for small-area estimation. Recent efforts using BRFSS data demonstrate the usefulness of such advanced methods to provide prevalence data on health factors for select metropolitan and micropolitan statistical areas (33).

Counties are the unit of measurement for the CHSI because most comparable data are not available below county level. However, as health disparities are increasingly understood and addressed, the demand for more detailed local information will grow. Advances in developing data for local areas can help communities identify core indicators to guide action, conduct trend analyses, and consider relevant qualitative data. The CHSI can benefit from these advances as well as contribute to their development.

### Sustaining CHSI

Public health programs are increasingly integrating quality improvement and innovative techniques into systems and activities designed to improve a community's health. The use of evidence — in particular, data about community health — is essential for making improvements. However, the progression from data to planning, action, and improvement is not always guaranteed. As the CHSI moves into its next phase, the development of training and tools for public health practice will take on increasing importance. For the CHSI to be sustained, its utility for improving community health must be demonstrated and become apparent to users and policy makers. The CHSI is developing case studies of selected communities that use local data to monitor health in order to develop and advocate sound policies, implement prevention strategies, and foster environments conducive to health. The CHSI plans to post these case studies on the CHSI Web site in the latter part of 2008. Also under development is an online training tool to assist the CHSI users with accessing and using the CHSI data and resources.

The future of the CHSI is also contingent on further development of the public–private partnerships that have been essential to the release of CHSI 2008. The CHSI Steering Committee, with support from RWJF, will engage in a series of planning sessions to explore the aforementioned strategies, as well as others, for improving and sustaining the CHSI.

## Conclusion

CHSI 2008 provides county-level health profiles for all 3141 U.S. counties to facilitate the examination of community health indicators that can be used to address community health. By bringing together data from multiple sources, the CHSI provides easy-to-understand reports that convey a wide range of public health issues and the uniqueness of local needs and assets. Learning how communities use the CHSI to initiate system and policy changes that improve health and increase public–private partnerships will enhance future versions of this resource.
